# The Regulation of Xylem Development by Transcription Factors and Their Upstream MicroRNAs

**DOI:** 10.3390/ijms231710134

**Published:** 2022-09-04

**Authors:** Pengfang Sun, Huilin Wang, Pan Zhao, Qiulin Yu, Yumei He, Wenhong Deng, Huihong Guo

**Affiliations:** 1 National Engineering Research Center of Tree Breeding and Ecological Restoration, College of Biological Sciences and Biotechnology, Beijing Forestry University, No. 35, Tsing Hua East Road, Haidian District, Beijing 100083, China; 2 Analytical and Testing Center, Beijing Forestry University, No. 35, Tsing Hua East Road, Haidian District, Beijing 100083, China

**Keywords:** xylem development, transcription factor gene, *HD-ZIP*, *MYB*, *NAC*, microRNAs, regulatory role

## Abstract

Xylem, as a unique organizational structure of vascular plants, bears water transport and supports functions necessary for plant survival. Notably, secondary xylem in the stem (i.e., wood) also has important economic and ecological value. In view of this, the regulation of xylem development has been widely concerned. In recent years, studies on model plants *Arabidopsis* and poplar have shown that transcription factors play important regulatory roles in various processes of xylem development, including the directional differentiation of procambium and cambium into xylem, xylem arrangement patterns, secondary cell wall formation and programmed cell death. This review focuses on the regulatory roles of widely and thoroughly studied *HD-ZIP*, *MYB* and *NAC* transcription factor gene families in xylem development, and it also pays attention to the regulation of their upstream microRNAs. In addition, the existing questions in the research and future research directions are prospected.

## 1. Introduction

**Figure 1:**
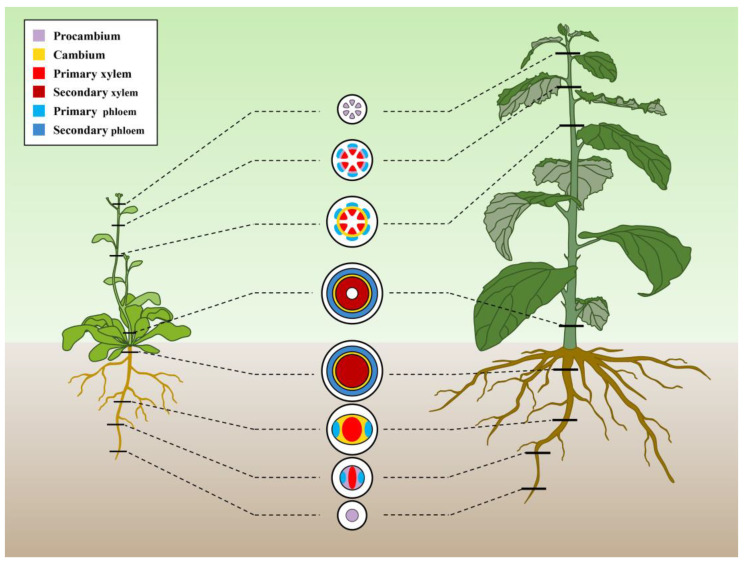
Schematic representation of xylem development in vascular plants represented by *Arabidopsis* and poplar.

Xylem is present in all vascular plants and is the main component of vascular tissue, which is mainly composed of tracheary elements such as vessels and fibers. Its main function is to transport water and inorganic salts and also to provide mechanical support [1]. Xylem includes primary xylem and secondary xylem, and its development involves two stages: primary growth and secondary growth [2,3]. In primary growth, i.e., elongation growth, the primary xylem is derived from the division and differentiation of procambium (Figure 1), which is divided into early differentiated protoxylem and late differentiated metaxylem. In secondary growth, i.e., thickening growth, the secondary xylem is produced by the division and differentiation of cambium (Figure 1). Notably, secondary xylem in the stem, also known as wood, is the main source of tree biomass, which can be used for multiple purposes, such as papermaking, furniture, construction and biofuels [4]. In addition, carbon storage in wood is also crucial for balancing the level of carbon dioxide in the atmosphere [5]. Therefore, research on the developmental regulation of xylem has been the focus and hotspot in the field of plant developmental biology, and it has received more and more attention in recent years.

**Figure 2:**
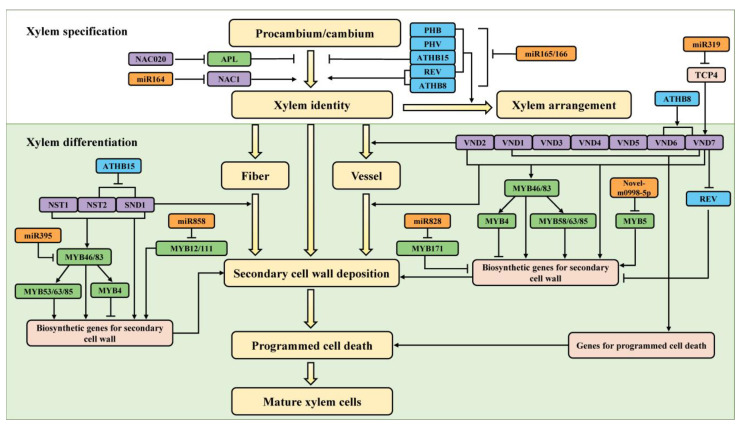
Genetic networks of xylem development regulated by *HD*-*Zip III*, *MYB* and *NAC* transcription factor genes and their upstream microRNAs. *HD*-*Zip III*, *MYB* and *NAC* genes are shown in blue, green and purple boxes, respectively. *HD*-*Zip III* genes are primarily involved in the differentiation of procambium or cambium into xylem and arrangement patterns of the xylem. *MYB* genes mainly regulate the development of xylem secondary cell walls. *NAC* genes chiefly participate in xylem vessel differentiation and secondary cell wall development. These three types of transcription factor genes are regulated by different upstream microRNAs. Black arrows represent activation, black lines with bars represent repression. The functions of most genes included in Figure 2 have been demonstrated in poplar.

The development of xylem begins with the division of procambium or cambium, followed by their directed differentiation into xylem (i.e., xylem specification) and a series of differentiation processes such as secondary cell wall formation and programmed cell death [6], which involve complex molecular regulatory networks. Accumulated data have shown that transcription factors play important regulatory roles in the establishment and development of xylem. Transcription factors are a class of proteins that regulate the expression of target genes by binding to their downstream target gene promoters [7]. In recent years, many key transcription factors regulating xylem development have been identified from model plants *Arabidopsis* and poplar [8,9]. These transcription factors belong to different families, among which HD-ZIP (homeo-domain leucine zipper), MYB (v-myb avian myeloblastosis viral oncogene homolog) and NAC [NAM (no apical meristem), ATAF (*Arabidopsis* transcription activation factor) and CUC (cup-shaped cotyledon)] family members play important roles in xylem development and have been extensively and thoroughly studied. With the deepening of research, it was found that genes encoding these transcription factors are regulated by their upstream MicroRNAs (miRNAs). miRNAs are a class of endogenous non-coding small RNAs of approximately 21–22 nucleotides in length, which usually repress the expression of their target genes at the post-transcriptional level through the cleavage or translational repression of their target mRNAs [10]. In the following sections, the regulatory roles of these three types of transcription factor genes and their upstream miRNAs in xylem development are reviewed and discussed in detail (Figure 2).

## 2. Regulation of Xylem Development by Transcription Factors

### 2.1. HD-Zip Gene Family

HD-ZIP is a class of plant-specific transcription factors. According to the homology of its domains, the characteristics of its gene structure and other motifs, the HD-ZIP family can be divided into four subfamilies: HD-Zip I, HD-Zip II, HD-Zip III and HD-Zip IV. Among these four subfamilies, the HD-Zip III subfamily plays a much more important role in the regulation of xylem development than the other three subfamilies, and its research is also more extensive. The HD-Zip III subfamily has five members in *Arabidopsis*, namely *Arabidopsis thaliana homeobox 8* (*ATHB8*), *Corona* (*CAN*)/*ATHB15*, *Revoluta* (*REV*), *Phabulosa* (*PHB*)/*ATHB14* and *Phavoluta* (*PHV*)/*ATHB9* [11,12]. The five members function individually or synergistically in the directional differentiation, arrangement pattern and secondary cell wall formation of xylem (Figure 2).

*ATHB8* overexpression promotes the differentiation of procambium and cambium into primary and secondary xylem in the roots and stems of transgenic *Arabidopsis* earlier than it does in the wild type [13,14]. In poplar, the overexpression of *PtrHB7*, the homolog of *ATHB8*, produces more secondary xylem cells and fewer secondary phloem cells in the stem, and this phenotype is more pronounced with higher *PtrHB7* expression levels [15], indicating that *PtrHB7* promotes the differentiation of cambium into xylem but inhibits its differentiation into phloem and that it regulates the homeostasis between secondary xylem and phloem tissues depending on its expression abundance. In *Arabidopsis*, the overexpression of antisense *ATHB15* results in a dramatic expansion of both primary and secondary xylem in the stem [16,17]. In poplar, the up-regulation of *POPCORONA* (*PCN*), the homolog of *ATHB15* (*CNA*), leads to delayed secondary xylem differentiation in the stem [18,19]. These findings indicate that *ATHB8* and *PtrHB7* are positive regulators for xylem specification, whereas *ATHB15* and *PCN* are negative regulators. Moreover, the regulation of xylem specification by *ATHB8*/*PtrHB7* and *ATHB15*/*PCN* in *Arabidopsis* and poplar appear to be different. *ATHB8* temporally advances xylem specification in *Arabidopsis*, and its homolog *PtrHB7* promotes xylem specification in poplar by enhancing the directed differentiation of cambium into xylem. In contrast, *ATHB15* inhibits xylem differentiation in *Arabidopsis* and temporally delays xylem specification in poplar. *ATHB15*/*PCN* is also involved in the developmental regulation of the secondary cell wall. In *Arabidopsis*, the *athb15*/*cna* mutant results in the abnormal lignification of pith cells [20,21], and the down-regulation of its ortholog *PCN* produces a similar phenotype in poplar [18,22]. *REV*, another member of the *HD*-*Zip III* subfamily, and its homolog participate in regulating the differentiation of cambium into xylem and the arrangement pattern of xylem as well as the formation of the secondary cell wall. In *Arabidopsis*, *REV* mutation leads to a reduction in the number of tracheary elements and the disappearance of fibers in the secondary xylem of the stem [23,24], and the up-regulation of *REV* expression results in the transformation of normal collateral bundles into amphivasal bundles [25,26]. In poplar, the overexpression of the *REV* homolog, *popREVOLUTA* (*PRE*), leads to the up-regulation of lignification-related genes and produces additional secondary xylem in the cortex of the stem [27,28]. Besides *REV*, the other three members, *PHB*, *PHV* and *ATHB15,* have also been shown to be involved in regulating the arrangement pattern of vascular tissues, including xylem. The *phb phv rev* triple mutant produces abnormal amphicribral bundles in the stem [29]. *REV* is primarily responsible for the arrangement pattern of xylem, whereas *PHB* and *PHV* mainly contribute to the arrangement pattern in lateral organs [25,29,30], indicating that *REV* plays a more important role than *PHB*/*PHV* in regulating xylem arrangement patterns. In contrast to the *phb phv rev* triple mutant, both antisense *ATHB15* overexpression plants and the *phb phv athb15* triple mutant produce abnormal amphivasal bundles in the stem [16,31], suggesting that *REV* and *ATHB15* have opposite effects in regulating vascular patterns.

### 2.2. MYB Gene Family

The MYB family, as one of the largest transcription factor families in plants, was initially found to be involved in the stress response of plants and was later found to play an important role in the regulation of xylem development [32,33]. MYB transcription factors mainly participate in regulating the secondary cell wall formation during xylem differentiation, constituting a complex regulatory network with MYB46 and MYB83 as the main switches (Figure 2). These two MYB transcription factors can activate the expression of a series of downstream genes by combining the secondary cell wall MYB response elements in their target gene promoters to regulate the development of the secondary cell wall [34]. In *Arabidopsis*, the overexpression of *MYB46* and *MYB83* up-regulate the expression levels of genes related to the synthesis of cellulose, xylan and lignin, the main components of the secondary cell wall, resulting in the thickening of the secondary cell wall in the stem. T-DNA mutation analysis found that *MYB46* and *MYB83* are functionally redundant, and double T-DNA knockout mutations of *MYB83* and *MYB46* lead to a loss of the secondary cell wall in vessels [34,35,36]. Poplar *PtrMYB2*, *PtrMYB3*, *PtrMYB20* and *PtrMYB21* are homologs of *Arabidopsis MYB46* and *MYB83*, and the defects that the secondary cell wall cannot be thickened and that growth arrest is present in the *myb46 myb83* double mutant were rescued by their overexpression [34,37,38,39]. These findings show that *PtrMYB2*/*3*/*20*/*21* genes function redundantly in xylem secondary cell wall formation and also indicate that, from *Arabidopsis* to poplar, the copies of the *AtMYB46*/*83* homologs have been significantly expanded, which may be related to the fact that the lignification degree of poplar is significantly higher than that of *Arabidopsis*.

*MYB46*/*83* target genes *MYB58*, *MYB63* and *MYB85* are transcriptional activators that regulate lignin biosynthesis in xylem differentiation [40,41]. In *Arabidopsis*, the down-regulation of *MYB58* and *MYB63* expression results in reduced lignin deposition and secondary cell wall thinning, whereas *MYB58*/*63* overexpression produces ectopic lignin deposition in the stem [42,43]. Both *MYB58*/*63* and its homolog *PtrMYB28* further activate the expression of downstream lignin biosynthesis-related genes [44]. These studies demonstrate that *MYB58*/*63* and its homologous gene are functionally conserved in *Arabidopsis* and poplar. However, some functional differentiation occurrs between *MYB85* and its poplar homologous gene. In *Arabidopsis*, the overexpression of *MYB85* promotes lignin biosynthesis, leading to the ectopic deposition of lignin in the stem [45], whereas in poplar, the overexpression of the *AtMYB85* homolog *PtoMYB92* not only results in the ectopic deposition of lignin but also produces more secondary xylem cells in the stem [46]. These findings suggest that *AtMYB85* only regulates the development of the secondary cell wall, and its homolog *PtoMYB92* not only regulates the development of the secondary cell wall but also the differentiation of cambium into xylem. *MYB4*, another target gene of *MYB46*/*83*, is a negative regulator of lignin biosynthesis. In *Arabidopsis*, *MYB4* overexpression inhibits lignin biosynthesis and accumulation [47,48]. Poplar *PdMYB221* is a homologous gene of *AtMYB4*, and its overexpression in *Arabidopsis* results in the thinning of the xylem secondary cell wall in the stem [49]. *MYB46* and *MYB83* not only regulate the expression of downstream transcription factors, but they also regulate the expression of secondary cell wall biosynthesis-related enzyme genes. For example, *MYB46* can directly regulate the expression of cellulose synthase genes (*CESA4*/*CESA7*/*CESA8*) and xylan synthase genes (*FRA8*/*IRX8*/*IRX9*/*IRX14*), thereby regulating the formation of cellulose and xylan in the secondary cell wall [50,51].

In addition to the *MYB46*/*83*-based regulatory network, some other members of the *MYB* family are also involved in the regulation of xylem development. *MYB61* is a positive regulator of xylem differentiation. Mutation in *Arabidopsis MYB61* results in reduced xylem vessels, secondary cell wall thinning and sometimes cytoplasmic retention in cells [52]. In poplar, the overexpression of the *AtMYB61* homolog *PtoMYB216* leads to the ectopic deposition of lignin and the thickening of the secondary cell wall in the xylem of the stem [53]. On the other hand, the *MYB* gene *ALTERED PHLOEM DEVELOPMENT* (*APL*) is a negative regulator of xylem specification. Its overexpression in *Arabidopsis* inhibits the directed differentiation of procambium into xylem but promotes the directed differentiation of phloem, and *APL* knockout leads to the formation of xylem-like tracheary elements in the phloem of the root [54,55]. Poplar *PaMYB199* is also a negative regulator of xylem development, and its overexpression leads to a reduction in the number of cambium and xylem cells and the thinning of the xylem secondary cell wall in the stem [56].

### 2.3. NAC Gene Family

Previous studies have shown that, like *MYB* genes, NAC transcription factors are mainly involved in the regulation of plant stress responses [57]. Later, it was also found that NAC transcription factors play an important regulatory role in xylem development [58,59].

During xylem development, a group of NAC transcription factors called SWNs (Secondary Wall NACs) are the top switches that activate the secondary cell wall biosynthetic network and are also involved in regulating the directed differentiation of vessels and programmed cell death (Figure 2) [60,61]. The members of SWNs include VNDs (vascular-related NAC domain: AtVND1 to AtVND7), NST1 (NAC secondary wall thickening promoting factor1), NST2 and SND1 (secondary wall-associated NAC domain1) [61]. The homologs of these members are named WNDs (for wood-associated NAC domain transcription factors) [62,63] or VNSs (for VND, NST/SND-, SOMBRERO-related proteins) [64] or PtrSNDs/PtrVNDs in poplar [60,65] (Supplementary Material S1, Table S1).

Among seven members of the VNDs, VND6 and VND7 were the first to be identified as key transcription factors involved in vessel differentiation in the root of *Arabidopsis*. Both *VND6* and *VND7* overexpression lead to the transdifferentiation of xylem parenchyma cells into tracheary elements in the root and induce the expression of genes related to secondary cell wall biosynthesis and programmed cell death. The secondary cell wall of vessels produced by *VND6* overexpression have reticulate and pitted thickening similar to those of metaxylem vessels, whereas the secondary cell wall of vessels produced by the *VND7* overexpression present annular and spiral thickening, similar to those of protoxylem vessels. Conversely, the down-regulation of *VND6* expression inhibits metaxylem vessel formation but not protoxylem vessel formation, whereas the down-regulation of *VND7* expression inhibits protoxylem vessel formation but not metaxylem vessel formation [66,67,68]. Subsequent studies have revealed that, besides regulating secondary cell wall deposition and programmed cell death, *VND1*-*5* also participates in the regulation of vessel differentiation. In *Arabidopsis*, the overexpression of *VND1*-*5* leads to the up-regulation of genes related to secondary cell wall formation and the secondary cell wall thickening of vessels and parenchyma cells in the stem. The overexpression of *VND1*/*3*/*4*/*5* activates the expression of genes related to programmed cell death (except for *VND2*) [69,70]. The *vnd1 vnd2 vnd3* triple mutant inhibits the differentiation of xylem vessels in cotyledons [71]. *VND2* and *VND3* promote the differentiation of metaxylem cells in roots [72]. In poplar, the overexpression of the *AtVND4*/*5* homolog *PdWND3A* increases xylem vessels and lignin content in the stem [73]. The above-mentioned findings indicate that the functions of the seven members of *VNDs* are relatively conserved in xylem differentiation. However, their functional divergences also emerge to some extent. For example, *VND6* and *VND7* specifically regulate the vessel differentiation and development of metaxylem and protoxylem, respectively, and *VND2* is not involved in the regulation of xylem programmed cell death processes like other members.

*SND1*, *NST1* and *NST2* mainly regulate the deposition of fiber secondary cell walls during xylem differentiation. The down-regulation of *SND1* expression leads to the thinning of fiber secondary cell walls in the stem of *Arabidopsis*, whereas *SND1* overexpression leads to the ectopic deposition of the secondary cell walls in fibers and parenchyma cells [74,75]. In poplar, *PtrWND1B*, the homolog of *AtSND1*, can be selectively spliced to generate a short transcript (*PtrWND1B*-*s*) and a long transcript (*PtrWND1B*-*l*). *PtrWND1B*-*s* overexpression gives rise to the thickening of fiber secondary cell walls in the stem, and *PtrWND1B*-*l* overexpression results in secondary cell wall thinning. However, *PtrWND1B* overexpression has no significant effect on the formation of fiber secondary cell walls [75,76], suggesting that the two spliced variants of *PtrWND1B* may antagonize each other and jointly regulate the deposition of fiber secondary cell walls. It is worth noting that, in *Arabidopsis*, a single mutation of either *SND1* or *NST1* does not cause changes in the thickness of fiber secondary cell walls in the stem [77,78,79]. The *snd1 nst1* double mutant results in the loss of fiber secondary cell walls, but varying degrees of the thickening of interfascicular fiber secondary cell walls are still present in older stems [77,80,81]. The *snd1 nst1 nst2* triple mutant leads to a complete loss of the secondary cell wall in both fibers and interfascicular fibers [80]. In poplar, a quadruple mutant of *Pt* × *tVNS9*–*12*, which is the homolog of *AtSND1* and *AtNST1*/*2*, shows a thinner secondary cell wall in xylem parenchyma cells and in fibers of the stem compared with the wild type [82]. These studies show that *SND1*/*NST1*/*NST2* and their homologous genes in poplar are involved in regulating the formation of fibers or interfascicular fiber secondary cell walls, among which *SND1* and *NST1* primarily regulate the formation of fiber secondary cell walls, and *NST2* mainly regulates the formation of interfascicular fiber secondary walls.

Interestingly, it was found that *NAC* genes can interact with *MYB* and *HD-Zip III* genes to regulate xylem development. All members of SWNs are involved in the regulation of secondary cell wall biosynthesis by directly targeting *MYB46*/*83* [45,61,69,83]. SWN member *VND7* can also promote lignin biosynthesis by inhibiting the expression of *HD*-*Zip III* gene *REV* [84]. On the other hand, the *HD*-*Zip III* gene *ATHB15* regulates secondary cell wall development by inhibiting the expression of SWN members, *SND1* and *NST2* [20], and *ATHB8* regulates vessel differentiation by promoting the expression of *VND6* and *VND7* [85]. In addition to SWN genes, *NAC020* of the *NAC* family was also found to interact with *MYB* to participate in xylem development. *NAC020* is an upstream negative regulator of *APL*, a member of the *MYB* family, and the overexpression of the *NAC020* in *Arabidopsis* causes partial discontinuity of *APL* expression in the root, thereby resulting in discontinuous vessel differentiation [55,86]. Given that *APL* is a suppressor of xylem differentiation, it has been suggested that *NAC020* can promote xylem vessel differentiation by inhibiting the expression of *APL*.

## 3. Upstream miRNA Regulation of the *HD-Zip III*/*MYB*/*NAC* in Xylem Development

*HD*-*ZIP III* genes are regulated by miR165/166 (Supplementary Material S2, Figure S1), and the five members of *HD*-*ZIP III* genes are conserved at the sequence of the miR165/166 binding site [87,88]. In *Arabidopsis*, the splicing site mutation of the miR165 target gene *REV* leads to the up-regulation of *REV*, the conversion of normal collateral bundles to amphivasal bundles and the thickening of fiber secondary cell walls in the stem [25]. The overexpression of miR165a results in the down-regulation of all *HD*-*ZIP III* genes, thereby reducing the number of xylem cells and thinning the fiber secondary cell walls in the stem [89,90], whereas the overexpression of antisense miR165a leads to a shift in the xylem arrangement from collateral bundles to amphivasal bundles [91]. The overexpression of miR165b, another member of the miR165 family, results in significant down-regulation in *PHB*, *PHV* and *AtHB15* expression and in slight down-regulation in *REV* expression, whereas *ATHB8* expression is unaffected, resulting in abnormal amphivasal bundles in the pith [20]. These data indicate that miR165a acts on all *HD*-*ZIP III* members and is involved in xylem differentiation, arrangement patterns and secondary cell wall development, and miR165b acts on *PHB*, *PHV* and *AtHB15* and only participates in the regulation of xylem arrangement patterns. MiR166 and miR165 differ by only one base in the mature region, and they jointly target the *HD*-*ZIP III* genes. In *Arabidopsis*, miR166a overexpression down-regulates the expression of *ATHB15*, *PHB*, *PHV* and *ATHB8*, but do not affect the expression of *REV*, resulting in the expansion of xylem in the stem [16,92]. The overexpression of miR166g results in the down-regulation of *ATHB15*, *PHB* and *PHV* but in the up-regulation of *REV* expression, whereas *ATHB8* expression is unaffected, resulting in additional xylem in the lateral phloem and abnormal amphivasal bundles in the pith [93,94]. The overexpression of miR166a and miR166g have the same effect on the expression of *ATHB15*, *PHB* and *PHV*, but their effects on the expression of *ATHB8* and *REV* are inconsistent, indicating that these two miR166 family members present functional divergence to some extent. In addition, the use of short tandem target mimic (STTM) technology can eliminate the inhibitory effect of miR165/166 on its target genes. STTM165/166 overexpression leads to the up-regulation of all *HD*-*ZIP III* genes and xylem expansion, but the arrangement of xylem changes from collateral bundles to amphivasal bundles in the stem [95,96].

MiRNAs are involved in the regulation of secondary cell wall component biosynthesis by targeting *MYB* genes during xylem differentiation (Supplementary Material S3, Figure S2). In *Arabidopsis*, the overexpression of miR858a down-regulates the expression of its target genes *MYB11*, *MYB12* and *MYB111*, giving rise to the down-regulation of flavonoid biosynthesis-related genes and to the up-regulation of lignin biosynthesis-related genes, thereby resulting in an increase in xylem lignin content. Conversely, the use of an miRNA target mimic (MIM) up-regulates the expression levels of miR858a target genes *MYB11*, *MYB12* and *MYB111*, giving rise to the up-regulation of flavonoid biosynthesis-related genes and to the down-regulation of lignin biosynthesis-related genes, thereby resulting in a decrease in lignin content in the stem [97]. There is substrate competition between flavonoid biosynthesis and lignin biosynthesis in the phenylpropane metabolic pathway [97], suggesting that miR858a-*MYB11*/*12*/*111* regulatory modules can promote lignin biosynthesis by inhibiting flavonoid biosynthesis. In poplar, the overexpression of miR828 down-regulates the expression of its target genes, *MYB11* and *MYB171*, and further inhibits the expression of lignin biosynthesis-related genes, resulting in a decrease in the number of xylem cells and lignin content, as well as in the thinning of the secondary cell wall in the stem, and the overexpression of STTM828 shows the phenotype opposite to miR828 overexpression [98]. These data reveal that miR858a promotes lignin biosynthesis by inhibiting the expression of *MYB11*/*12*/*111*, whereas miR828 inhibits lignin biosynthesis by down-regulating the expression of *MYB11*/*171*, and these two miRNAs play opposite regulatory roles in xylem differentiation. In view of the above-mentioned same effects of miR858a and miR828 on the expression of *MYB11*, it is speculated that miR858a promotes lignin biosynthesis by primarily targeting *MYB12*/*111*, whereas miR828 inhibits lignin biosynthesis by mainly targeting *MYB171*. Moreover, in poplar, a new miRNA (Novel-m0998-5p) was found to be involved in xylem differentiation in the stem by targeting *MYB5*, and the targeted *MYB5* could activate the expression of the key lignin biosynthesis gene *PAL* (Phenylalanine ammonia-lyase) by forming the MYB-bHLH-WDR complex to promote lignin biosynthesis [99]. Recently, miR395c was found to participate in the biosynthesis of several main components of the secondary cell wall by targeting *MYB46* in poplar. MiR395c overexpression down-regulated the expression of *MYB46,* thereby inhibited the expression of lignin, hemicellulose, and cellulose biosynthesis-related genes, resulting in the thinning of fiber secondary cell walls in the stem [100].

Currently, the research on miRNAs directly regulating *NAC* genes to participate in xylem development is very limited (Supplementary Material S4, Figure S3). It was only found that miR164 may be involved in the xylem specification regulation by directly targeting *NAC1*. In *Arabidopsis*, the up-regulation of miR164 results in the down-regulation of its target gene *NAC1,* whereas the down-regulation of miR164 results in the up-regulation of *NAC1*. Moreover, it was found that the overexpression of *NAC1* leads to stem thickening [101,102]. Given that stem thickening is mainly related to xylem expansion [103], it has been suggested that, for secondary xylem specification, miR164 is a negative regulator, whereas *NAC1* is a positive regulator. After the transfection of poplar *_Pro_*peumiR164b::*GUS* and *_Pro_*PeuNAC070::*GUS* into *Arabidopsis*, it was found that peu-miR164b is only expressed in primary stems, that the *NAC1* homolog *PeuNAC070* is expressed in both the primary and secondary stem and that the overexpression of *PeuNAC070* inhibits stem elongation [104]. These results suggest that *NAC1*/*PeuNAC070* promoting stem thickening but inhibiting stem elongation may be related to the fact that miR164 is only expressed in primary stems, because the expression of *NAC1*/*PeuNAC070* is inhibited by miR164 in primary stems but not regulated by miR164 in secondary stems. Subsequent studies have shown that miR319 could also participate in the regulation of xylem differentiation by indirectly regulating the *NAC* transcription factor (Supplementary Material S4, Figure S3). In *Arabidopsis* and poplar, miR319a indirectly regulates the expression of *NAC* family member *AtVND7* and its homolog *PtoWND6A*/*6B* by targeting *AtTCP4* of the *TCP* (*TEOSINTE BRANCHED1*/*CYCLOIDEA*/*PCF*) family and its homolog *PtoTCP20*, respectively, thereby regulating the development of xylem. MiR319a overexpression leads to the down-regulation of its target genes *AtTCP4* and *PtoTCP20*, which triggers the down-regulation of *AtVND7* and *PtoWND6A*/*6B*, resulting in decreased vessel elements and the thinning of vessel secondary cell walls in the root [105,106]. This finding indicates that miR319a negatively controls the differentiation of procambium and cambium into xylem and secondary cell wall development by *NAC-*mediated regulation.

## 4. Conclusions and Prospective

*HD*-*Zip III*, *MYB* and *NAC* transcription factors have been confirmed to play partially or completely different regulatory roles in various processes of xylem development (Figure 2). *HD*-*Zip III* genes are primarily involved in the differentiation of procambium or cambium into xylem and arrangement patterns of the xylem. Among five members of *HD*-*Zip III*, *REV*, *ATHB8* and *ATHB15* promote xylem differentiation, and *REV* and *ATHB15* also co-operate with *PHB* and *PHV* to regulate the arrangement pattern of xylem. *MYB* genes mainly regulate the formation of xylem secondary cell walls. In the *MYB* family, a molecular network is formed that mainly uses *MYB46* and *MYB83* as the main switch to regulate xylem development, and they regulate the formation of the secondary cell wall by targeting *MYB4*, *MYB58*, *MYB63* and *MYB85*. *NAC* genes, which chiefly participate in xylem vessel differentiation and secondary cell wall development. In the *NAC* family, a molecular network with SWNs as top switches is formed to regulate the development of xylem. *VNDs*, as the main members of SWNs, primarily regulate the differentiation of vessel and the formation of the secondary cell wall, and the other members, *NST1*, *NST2* and *SND1,* mainly regulate the deposition of fiber secondary walls. *HD*-*Zip III*, *MYB* and *NAC* transcription factor gene families are also regulated by their upstream miRNAs (Figure 2). The *HD*-*ZIP III* genes are directly regulated by miR165/166 and are involved in the development of xylem. In the *MYB* family, *MYB12*/*111* and *MYB171* are directly regulated by miR858 and miR828, respectively, whereas *MYB11* is jointly regulated by both miR858 and miR828, which all participate in the biosynthesis of lignin. In addition, *MYB46* and *MYB5* are directly regulated by miR395 and Novel-m0998-5p, respectively, which also participate in lignin biosynthesis. In the *NAC* family, *NAC1* is directly regulated by miR164 and may be involved in regulating the secondary growth of xylem, and *VND7* is indirectly regulated by miR319, which is involved in xylem differentiation and secondary cell wall development.

Since *HD*-*ZIP III*, *MYB* and *NAC* genes all play important roles in xylem development, it is likely that there is a close interaction between them, but so far, only a few studies on their coordinated regulation of xylem development have been reported. Moreover, the upstream regulatory mechanisms of these three types of transcription factor genes, especially their regulation by upstream miRNAs, are still poorly understood. For example, different members of the miRNA family may have functional differentiation and accordingly differentially regulate their target genes, and the resulting regulatory mechanism is not very clear. To address these questions, related studies carried out in model plants and in other plant species in the future will help to better understand and reveal the molecular regulation mechanism of xylem development in vascular plants.

## Supplementary Materials

The supporting information can be downloaded at: https://www.mdpi.com/article/10.3390/ijms231710134/s1.

## Abbreviations

APL	Altered phloem development
ATHB	*Arabidopsis thaliana* homeobox
CESA	Cellulose synthase A
CNA	Corona
FRA	Fragile fiber
HD-ZIP III	Homeodomain leucine zipper class III
IRX	Irregular xylem
MIM	MicroRNA target mimic
miRNA	MicroRNA
MYB	V-myb avian myeloblastosis viral oncogene homolog
NAC	NAM (no apical meristem)/ATAF (*Arabidopsis* transcription activation factor)/CUC (cup-shaped cotyledon)
NST	NAC secondary wall thickening promoting factor
PAL	Phenylalanine ammonia-lyase
PaMYB	*Populus alba* × *Populus glandulosa* v-myb avian myeloblastosis viral oncogene homolog
PCN	Popcorona
PdMYB	*Populus* deltoides v-myb avian myeloblastosis viral oncogene homolog
PdWND	*Populus* deltoides for wood-associated NAC domain transcription factor
PeuNAC	*Populus* euphratica NAC
PHB	Phabulosa
PHV	Phavoluta
PRE	Poprevoluta
Pt × tVNS	*Populus tremula* × *Populus tremuloides* for VND, NST/SND-, SOMBRERO-related proteins
PtoMYB	*Populus tomentosa* v-myb avian myeloblastosis viral oncogene homolog
PtoTCP	*Populus tomentosa* teosinte branched1/cycloidea/pcf
PtoWND	*Populus tomentosa* for wood-associated NAC domain transcription factors
PtrHB	*Populus trichocarpa* homeobox
PtrMYB	*Populus trichocarpa* v-myb avian myeloblastosis viral oncogene homolog
PtrSND	*Populus trichocarpa* secondary wall-associated NAC domain
PtrVND	*Populus trichocarpa* vascular-related NAC domain
PtrWND	*Populus trichocarpa* for wood-associated NAC domain transcription factors
REV	Revoluta
SND	Secondary wall-associated NAC domain
STTM	Short tandem target mimic
SWN	Secondary wall NAC
TCP	Teosinte branched1/cycloidea/pcf
VND	Vascular-related NAC domain
VNS	For VND, NST/SND-, SOMBRERO-related protein
WDR	WD repeat
WND	For wood-associated NAC domain transcription factor
