# Supplementary material for: The Regulation of Xylem Development by Transcription Factors and Their Upstream MicroRNAs
Source: Int J Mol Sci. 2022 Sep 4;23(17):10134. doi: 10.3390/ijms231710134 (PMC9456210; doi:10.3390/ijms231710134)
Supplement: Supplementary file 1 [file ijms-23-10134-s001.zip › Supplementary Material S1.pdf]

Supplementary Material 1

Table S1. Putative *Populus trichocarpa* orthologs of *Arabidopsis* SWN transcription factors known to be involved in xylem development.

Arabidopsis SWN transcription factors		Putative poplar co-orthologs	References
NST	SND1/NST3 (ANAC012)	PtVNS11/PtrWND1B/PtrSND1-A2/PtrSND1-2 (Potri.001G448400)	Ohtani et al., 2011 [64]; Zhong et al., 2010a [62], 2010b [63]; Li et al. 2012 [65]; Johnsson et al., 2019 [60]
		PtVNS12/PtrWND1A/PtrSND1-A1/PtrSND1-1 (Potri.011G153300)	
	NST1	PtVNS9/PtrWND2A/PtrSND1-B1/PtrNST1-1 (Potri.014G104800)	
	NST2	PtVNS10/PtrWND2B/PtrSND1-B2/PtrNST1-2 (Potri.002G178700)	
VND	VND1 (ANAC037)	PtVNS01/PtrWND5A/PtrVND6-C1/PtrVND3-1 (Potri.007G014400)	Ohtani et al., 2011 [64]; Zhong et al., 2010a [62], 2010b [63]; Li et al., 2012 [65]; Johnsson et al., 2019 [60]
	VND2(ANAC076)	PtVNS02/PtrWND5B/PtrVND6-C2/PtrVND3-2 (Potri.005G116800)	
	VND3 (ANAC105)		
	VND4 (ANAC007)	PtVNS03/PtrWND4A/PtrVND6-B2/PtrVND6-3 (Potri.001G120000)	
	VND5 (ANAC026)	PtVNS04/PtrWND4B/PtrVND6-B1/PtrVND6-4 (Potri.003G113000)	
		PtVNS05/PtrWND3A/PtrVND6-A1/PtrVND6-1 (Potri.015G127400)	
		PtVNS06/PtrWND3B/PtrVND6-A2/PtrVND6-2 (Potri.012G126500)	
	VND6 (ANAC101)		
	VND7 (ANAC030)	PtVNS07/PtrWND6A/PtrVND7-1(Potri.013G113100)	Ohtani et al., 2011 [64]; Zhong et al., 2010a [62], 2010b [63]; Johnsson et al., 2019 [60]
		PtVNS08/PtrWND6B/PtrVND7-2(Potri.019G083600)	
