# Supplementary material for: The Regulation of Xylem Development by Transcription Factors and Their Upstream MicroRNAs
Source: Int J Mol Sci. 2022 Sep 4;23(17):10134. doi: 10.3390/ijms231710134 (PMC9456210; doi:10.3390/ijms231710134)
Supplement: Supplementary file 1 [file ijms-23-10134-s001.zip › Supplementary Material S2.pdf]

Supplementary Material S2

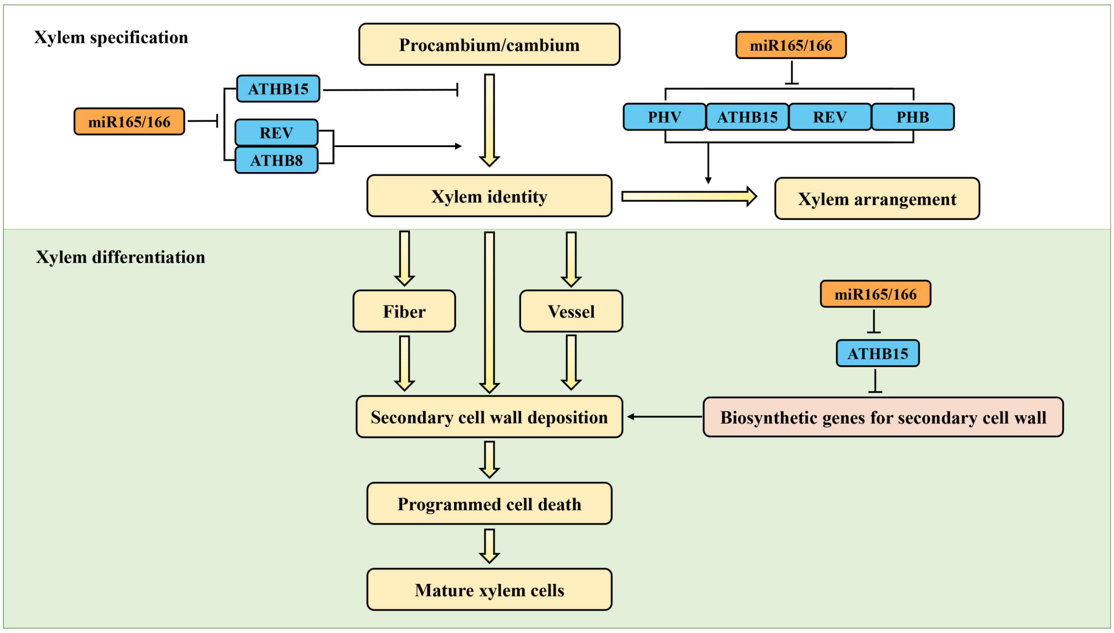

Figure S1. Genetic networks of xylem development regulated by *HD-Zip III* transcription factor genes and their upstream microRNAs. *HD-Zip III* genes are shown in blue boxes and they are primarily involved in the differentiation of procambium or cambium into xylem and arrangement pattern of the xylem. Black arrow represents activation, black line with a bar represents repression. The functions of most genes included in Figure S1 have been demonstrated in poplar.
