# Supplementary material for: The Regulation of Xylem Development by Transcription Factors and Their Upstream MicroRNAs
Source: Int J Mol Sci. 2022 Sep 4;23(17):10134. doi: 10.3390/ijms231710134 (PMC9456210; doi:10.3390/ijms231710134)
Supplement: Supplementary file 1 [file ijms-23-10134-s001.zip › Supplementary Material S4.pdf]

Supplementary Material S4

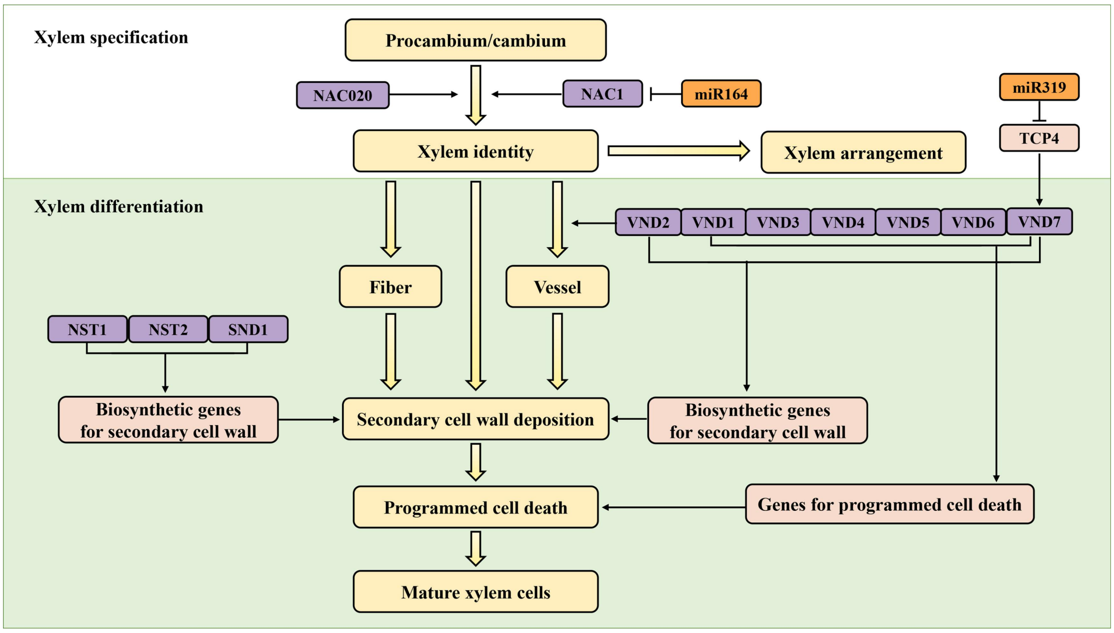

Figure S3. Genetic networks of xylem development regulated by *NAC* transcription factor genes and their upstream microRNAs. *NAC* genes chiefly participate in xylem vessel differentiation and secondary cell wall development. Black arrow represents activation, black line with a bar represents repression. The functions of most genes included in Figure S3 have been demonstrated in poplar.
